# Prostaglandin E2 modulates contractility of mouse bladder smooth muscle cells

**DOI:** 10.3389/fphys.2026.1910107

**Published:** 2026-08-12

**Authors:** Rok Šumak, Igor But, Andraž Stožer, Jurij Dolenšek, Tamara Serdinšek

**Affiliations:** 1 Department of General Gynaecology and Urogynaecology, Clinic for Gynaecology and Perinatology, University Medical Centre Maribor, Maribor, Slovenia; 2 Gynecology Clinic But, UGB, Maribor, Slovenia; 3 Institute of Physiology, Faculty of Medicine, University of Maribor, Maribor, Slovenia

**Keywords:** calcium, confocal imaging, detrusor muscle, prostaglandin E_2_, signaling, smooth muscle cell

## Abstract

**Background:**

Prostaglandin E2 (PGE_2_) is an important local mediator in the urinary bladder, where it contributes to the regulation of detrusor excitability and is implicated in pathological conditions such as overactive bladder. Although previous studies have shown that exogenous PGE enhances bladder contractile activity, its effects on calcium signaling in individual detrusor smooth muscle cells (SMCs) and its interaction with cholinergic stimulation remain incompletely understood.

**Methods:**

Acute detrusor tissue slices were prepared from adult female NMRI mice and loaded with the calcium indicator Calbryte 520 AM. Confocal calcium imaging was used to quantify spontaneous and stimulated intracellular Ca^2+^ activity in individual SMCs. The effects of PGE_2_ alone and in combination with increasing concentrations of carbachol (CCh) were assessed by measuring oscillation active time, frequency, and duration of Ca²^+^ events. Tissue contractions were evaluated by visual inspection of image sequences.

**Results:**

PGE_2_ increased spontaneous Ca^2+^ activity in detrusor SMCs, as reflected by a significant increase in active time due to increases in oscillation frequency, while oscillation duration remained unchanged. PGE_2_ also recruited additional SMCs into an active state and induced localized tissue contractions. In the presence of PGE_2_, lower concentrations of CCh evoked greater Ca²^+^ activity than under control conditions, suggesting that PGE_2_ increased baseline detrusor excitability and reduced the additional cholinergic input required to evoke detectable Ca²^+^ activity. This effect was again mediated predominantly by an increase in oscillation frequency rather than duration. At the tissue level, PGE_2_ enhanced CCh-induced contractility and shifted generalized contractions to lower CCh concentrations.

**Conclusions:**

PGE_2_ acts as a local excitatory modulator of mouse detrusor smooth muscle, increasing spontaneous Ca^2+^ activity and sensitizing the tissue to cholinergic stimulation. These effects are associated primarily with frequency-based modulation of Ca^2+^ oscillations and may represent a cellular mechanism contributing to detrusor overactivity under conditions of increased prostaglandin signaling.

## Introduction

The urinary bladder’s function in humans depends on tightly and complexly regulated detrusor smooth muscle activity to store and eject urine. During voiding, parasympathetic nerve impulses release acetylcholine (ACh) onto muscarinic receptors (primarily M2 and M3) in the detrusor, triggering an increase in intracellular Ca²^+^ and causing contraction of the bladder wall (Andersson, 2017). Conversely, during the filling phase, β_3_-adrenoceptor activation contributes to detrusor relaxation, as demonstrated in isolated human bladder preparations (Igawa et al., 1998; Takeda et al., 1999). In addition to neural control, the bladder exhibits intrinsic spontaneous activity modulated by local signals, so called non-adrenergic non-cholinergic (NANC) mechanisms. The urothelium senses mechanical stretch and releases ATP, which contributes to signaling to bladder afferent nerves (Ferguson et al., 1997; Cockayne et al., 2000). Urothelium-derived mediators can modulate detrusor contractility and bladder afferent activity (Heppner et al., 2024).

Prostaglandins are among key local modulators of bladder function, with PGE_2_ in particular playing a prominent role. PGE_2_ is an eicosanoid produced via cyclooxygenase (COX) enzymes, and it is generated within the bladder wall in response to physiological stress and inflammation. Mechanical stretch can induce urothelial cyclooxygenase-2 expression, and the urothelium represents an important source of bladder prostanoids (Jerde et al., 2006). Once released, PGE_2_ can act on both smooth muscle cells (SMCs) and afferent nerves in the bladder. PGE_2_ modulates Aδ- and C-fiber bladder afferent activity and may thereby contribute to bladder overactivity (Kuga et al., 2016).

In the urinary bladder, exogenous PGE_2_ has an excitatory effect. Classic organ bath experiments demonstrated that PGE_2_ causes a concentration-dependent increase in detrusor muscle tone and increase of rhythmic phasic contractions (Kobayter et al., 2012). Confocal Ca²^+^ imaging of isolated bladder strips further showed that PGE_2_ significantly elevates the frequency of spontaneous Ca²^+^ transients in detrusor SMCs (Kobayter et al., 2012). These data indicate that PGE_2_ can directly stimulate detrusor myocytes, inducing and synchronizing their Ca²^+^ oscillatory activity to produce coordinated contractions.

Given PGE_2_’s dual role as an inflammatory mediator and SMCs modulator, it is not surprising that dysregulation of PGE_2_ pathways is implicated in various pelvic pathologies. In the lower urinary tract, PGE_2_ signaling is similarly linked to dysfunctional contractility and sensation. Overactive bladder (OAB), which is characterized by urgency, frequency, and detrusor overactivity, exemplifies a disorder in which PGE_2_ pathways are upregulated. Patients with OAB show elevated levels of PGE_2_ in their urine compared to healthy individuals (Kim et al., 2006) indicating enhanced prostaglandin turnover in the bladder wall. These effects are thought to result from PGE_2_ sensitizing bladder afferent nerves and directly stimulating the detrusor, effectively causing the bladder’s tendency to contract at lower volumes. These clinical correlations underscore the importance of understanding PGE_2_’s actions in the bladder, as they may inform new therapeutic strategies to treat lower urinary tract disorders and pelvic pain conditions.

**Figure 1:**
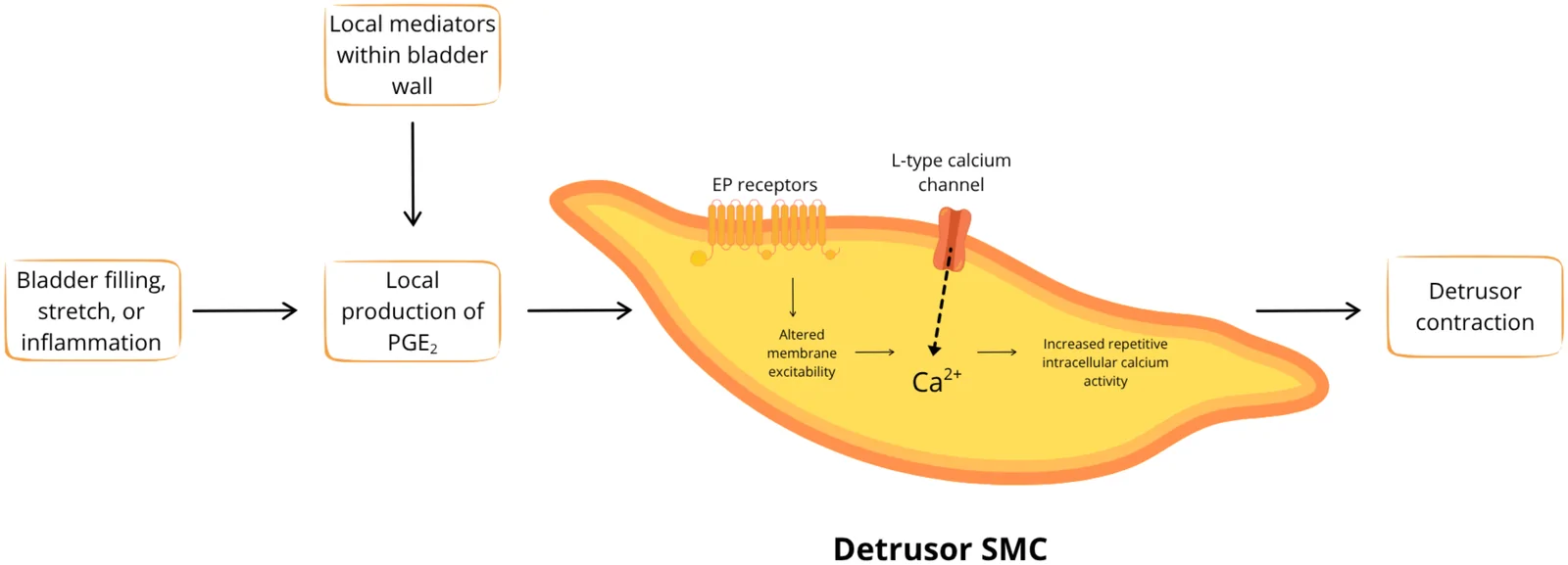
Proposed role of PGE_2_ in regulating detrusor smooth muscle excitability.

To place the present study in the context of existing knowledge, the principal primary studies investigating prostaglandin signaling, bladder smooth muscle physiology, urothelial regulation, and their relevance to bladder dysfunction are summarized in **Table 1**. The proposed role of PGE_2_ in regulating detrusor smooth muscle excitability is summarized in **Figure 1**.

**Table 1:** Comparative overview of primary studies investigating PGE_2_ signaling, urothelial regulation, bladder smooth muscle physiology, and relevance to OAB/UAB.

Study	Species/model	Experimental preparation	Main mechanism investigated	Key findings
(Anderson, 1982)	Rabbit	Isolated bladder smooth muscle	Direct effects of prostaglandins on detrusor contractility	Demonstrated that prostaglandins directly modulate bladder smooth muscle contractile responses.
(Schröder et al., 2004)	Mouse (WT and EP1−/−)	Conscious cystometry; intravesical PGE_2_; bladder outlet obstruction (BOO)	EP1 receptor contribution to PGE_2_-induced bladder overactivity	EP1 deficiency markedly reduced PGE_2_-induced and BOO-induced detrusor overactivity.
(Hindley et al., 2004)	Human (UAB patients)	Clinical study evaluating intravesical PGE_2_ therapy	Therapeutic effects of PGE_2_ in detrusor underactivity	PGE_2_ treatment produced only limited clinical improvement.
(Kim et al., 2006)	Human (OAB patients)	Urinary biomarker analysis	Clinical relevance of PGE_2_ in OAB	Urinary PGE_2_ concentrations were elevated in patients with OAB.
(Nile et al., 2010)	Guinea pig	Isolated bladder lamina propria	Prostaglandin-mediated urothelial/lamina propria signaling	Demonstrated functional prostaglandin signaling within bladder mucosal tissues.
(Kobayter et al., 2012)	Mouse	Isolated bladder strips; electrophysiology; confocal Ca²^+^ imaging; force recordings	PGE_2_ regulation of detrusor excitability	PGE_2_ increased spontaneous depolarizations, Ca²^+^ transient frequency, tone, and phasic contractions via EP receptors and L-type Ca²^+^ channels.
(Xue et al., 2013)	Mouse (EAE model)	*In vivo* voiding analysis; bladder biochemistry	EP3/EP4 receptor involvement in neurogenic bladder dysfunction	Increased bladder PGE_2_ and EP3/EP4 expression; receptor antagonists improved bladder dysfunction.
(Parajuli et al., 2014)	Guinea pig	Isolated detrusor smooth muscle; patch clamp; force recordings	BK-channel regulation by PGE_2_	PGE_2_ inhibited BK currents and increased detrusor excitability.
(Serdinšek et al., 2020)	Mouse	Acute detrusor tissue preparation; confocal Ca²^+^ imaging	CCh regulation of detrusor smooth muscle Ca²^+^ activity	Increasing CCh recruited additionalsmooth muscle cells and increased Ca²^+^ event activity.
(Stromberga et al., 2020)	Pig	Isolated bladder preparations	PGE_2_ modulation of urothelial and detrusor signaling	FP receptor antagonism attenuated PGE_2_- and PGF_2_α-induced contractile responses, indicating an important contribution of FP receptor signaling.

Bladder filling, mechanical stretch, and inflammation promote local production of prostaglandin E_2_ (PGE_2_) within the bladder wall. Based on previous studies, PGE_2_ is proposed to act through EP receptors to increase detrusor smooth muscle cell excitability, promote Ca²^+^ entry, and increase repetitive intracellular Ca²^+^ event activity, thereby facilitating detrusor contraction. The present study investigates the effects of exogenous PGE_2_ on Ca²^+^ event activity and cholinergic responsiveness in mouse detrusor smooth muscle cells. The illustrated signaling pathway is a conceptual summary based on previous literature and is not intended to imply that the underlying mechanisms were directly tested in the present study.

However, the cellular mechanisms linking PGE_2_ signaling to detrusor excitability remain incompletely defined. Although previous studies have shown that PGE_2_ increases spontaneous detrusor activity, it remains unclear how ongoing PGE_2_ exposure influences Ca²^+^ event activity in individual detrusor smooth muscle cells during cholinergic stimulation within preserved tissue architecture. Defining this interaction is important because prostaglandins and cholinergic signaling coexist in the urinary bladder and together regulate detrusor excitability under physiological and pathological conditions.

*To investigate these processes, we used an acute mouse detrusor slice preparation combined with confocal Ca²^+^ imaging, which enables quantitative analysis of Ca²^+^ event activity in individual detrusor smooth muscle cells in situ* (Serdinšek et al., 2020). Our aim was to determine whether ongoing PGE_2_ exposure alters spontaneous Ca²^+^ event activity and modifies responses to increasing concentrations of carbachol (CCh) in individual detrusor smooth muscle cells. Specifically, we investigated whether PGE_2_ enables lower concentrations of CCh to evoke detectable Ca²^+^ event activity under the present experimental conditions.

## Methods

Adult female NMRI mice were used in the experiments. Because the study was performed on animals, the National Medical Ethics Committee of the Republic of Slovenia determined that it was not competent to issue an approval for the study (reference no. 0120-349/2023/3). Animal use and euthanasia were approved by the Administration of the Republic of Slovenia for Food Safety, Veterinary Sector and Plant Protection (approval no. U34401-35/2018/2). All procedures were performed in accordance with applicable national and institutional guidelines.

### Tissue dissection

For tissue dissection, adult female NMRI mice were sacrificed by utilizing CO_2_ and cervical dislocation. For tissue collection, mice were placed individually in a non-prefilled glass chamber with an internal volume of approximately 2 L. The chamber was covered with a loosely fitted cardboard lid, and CO_2_ was introduced through tubing positioned near the centre of the chamber, with displaced gas escaping around the non-airtight lid. The CO_2_ flow was estimated to approximately 100% of the chamber volume per minute. Following respiratory arrest, cervical dislocation was performed as a secondary physical method to ensure death before tissue collection. Immediately thereafter, abdomen was accessed via lower median laparotomy and the bladder was dissected carefully. The dissected bladder was transferred into an ice-cold bathing physiological salt solution (PSS). PSS contained NaCl 118.4 mM, NaHCO_3_ 25.0 mM, NaH_2_PO_4_ 1.13 mM, CaCl_2_ 1.8 mM, KCl 4.7 mM, MgCl_2_ 1.3 mM, and glucose 11.1 mM, and was continuously gassed with carbogen (95% O_2_ and 5% CO_2_). All chemicals were from Sigma Aldrich. Subsequently, connective tissue and urothelium were gently removed from detrusor muscle under a light stereomicroscope (Nikon SMZ 745). The isolated detrusor muscle was cut into three to four tissue preparations (approximately 2–4 mm²) using sharp scissors. These preparations retained the full thickness of the detrusor muscle and are referred to throughout the manuscript as acute detrusor tissue slices, consistent with our previously published methodology (Serdinšek et al., 2020). Tissue slices were then loaded with the membrane–permeable calcium reporter dye Calbryte 520 AM (AAT Bioquest) final concentration 6.8 µM, with dimethyl sulfoxide (DMSO, 0.1% v/v) and Pluronic (Invitrogen, 0.4% v/v) in a PSS 30–40 minutes at room temperature on a shaker at 50 strokes per minute. We used an acute mouse detrusor slice preparation combined with confocal Ca²^+^ imaging, adapted from the previously validated *in situ* approach for studying detrusor smooth muscle cells described by Serdinšek et al. (2020).

### Confocal calcium imaging

Calcium imaging was performed immediately after dye incubation using an inverted confocal microscope (Leica TCS SP5 II). Calcium dye was excited at 492 nm and emitted fluorescence was detected with a HyD detector (range 500–600 nm). Calbryte 520 AM was used as a single-wavelength, intensity-based Ca²^+^ indicator. Fluorescence signals were not calibrated to absolute intracellular Ca²^+^ concentrations and are therefore expressed as relative fluorescence or arbitrary fluorescence units. The analysis was based on the detection and temporal characterization of Ca²^+^-dependent fluorescence events rather than on resting fluorescence or event amplitude. Images were acquired at 512 × 512 pixels at 1.90 Hz. For recordings, individual tissue slices were transferred to a recording chamber and perfused with PSS at 37 °C.

To investigate responses of the detrusor muscle to pharmacological stimuli, tissue slices were stimulated with prostaglandin E2 (PGE_2_). PGE_2_ was obtained from Tocris (Abingdon, UK), stored as stock solution in DMSO at -20 °C and diluted in PSS immediately before experimentation. In a pilot study we stimulated the slices with increasing concentration of PGE_2_ (0.1, 1, 10, and 50 µM), each stimulus for 10 minutes. The stimulation protocol consisted of a 5 min baseline perfusion with PSS, followed by 15 min of 50 µM PGE_2_ stimulation, and a final application of 50 µM CCh to confirm tissue responsiveness. Based on the pilot experiments and previous studies demonstrating that PGE_2_ in the micromolar range induces contractile responses in detrusor smooth muscle (Kobayter et al., 2012), 50 µM PGE_2_ was selected for all subsequent experiments. In separate set of experiments, we investigated response to increasing concentrations of the cholinomimetic drug carbamylcholine (carbachol, CCh) in PSS using a staircase-like protocol: PSS only 6 min, 0.1 µM CCh 6 min, 1 µM CCh 6 min, 10 µM CCh 6 min, and 25 µM CCh 6 min. The selected concentrations of CCh (0.1–25 µM) were chosen to cover a broad dose–response range from subthreshold to near-maximal effects, consistent with previously published protocols using cumulative cholinergic stimulation of detrusor smooth muscle (Serdinšek et al., 2020). In a separate set of experiments, 50 µM PGE2 was added to the CCh staircase stimulation.

### Data analysis

Obtained time series were analyzed off-line using custom scripts in Matlab software (The Mathworks, Inc, version R2024a). SMCs were identified according to their morphology and regions of interest (ROIs) corresponding to individual SMCs were manually outlined in Fiji (ImageJ version1.54p) to extract respective time series. These time series data were first corrected for bleaching using a fit consisting a combination of linear and exponential curve by utilizing custom made script in Matlab, and displayed as F/F_0_. We binarized individual Ca^2+^ oscillatory activity by detecting each peak using local maxima function in Matlab, and storing its beginning and end at half amplitude. This approach relied on manual setting of prominence for each recording, and enabled reliable detection of peaks for records with different amplitudes of peaks. Each binarization was displayed and visually confirmed. The binarized data was used to calculate oscillatory durations (rounded according to sampling frequency), frequencies (calculated as number of events per analysis interval), and active time (calculated as sum of oscillatory event durations relative to analysis interval) using custom made scripts in Matlab. Since SMCs are spontaneously active, we used active time to measure change in SMC activity following PGE_2_ stimulation using custom made scripts in Matlab: if active time increased, the SMC was labeled as activated by the stimulus. Lastly, tissue contraction following stimulation with PGE_2_ or CCh was determined visually by careful inspection of raw image sequence, and was visible as either local tissue contraction in case of PGE_2_ stimulation, or as global tissue contraction in case of CCh stimulation. For the contraction analysis, only preparations that retained responsiveness to the final carbachol (CCh) application were included, as this served as a criterion for tissue viability throughout the experiment. Data were quantified by expressing relative proportion of preparations exhibiting contraction. Quantified data between groups were compared using Mann-Whitney U test using GraphPad Prism (GraphPad Software, LLC, version 10.6.1).

## Results

To evaluate the functional effects of PGE_2_ on mouse bladder detrusor tissue, we first quantified spontaneous intracellular Ca^2+^ concentration ([Ca^2+^]_i_) activity in individual smooth muscle cells (SMCs) and assessed whether these responses were accompanied by tissue contractions. We next tested whether PGE_2_ modulates cholinergic responsiveness by comparing SMC activity and contractility during stimulation with increasing concentrations of carbachol (CCh) in the absence and presence of PGE_2_. .

### Effect of PGE_2_ on SMC activity

**Figure 2:**
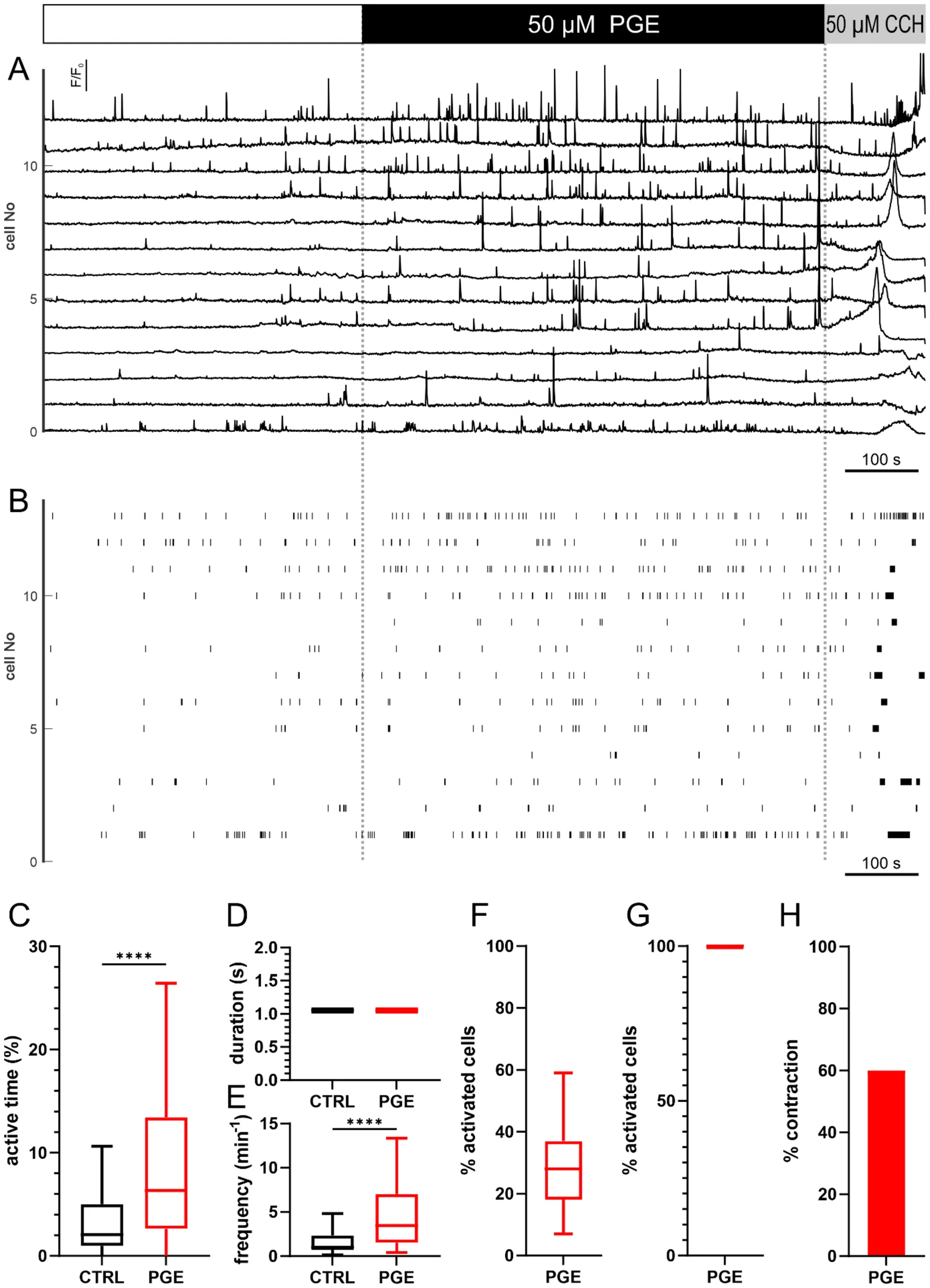
Effect of PGE_2_ on SMC activity. **(A, B)** Response of SMC from the same bladder to 50 µM PGE_2_ (raw data shown in panel A and binarized activity in panel **(B)**. Stimulatory protocol is indicated above traces. After each protocol we applied 50 µM CCh to confirm physiological response. **(C-E)** Active time **(C)**, duration **(D)** and frequency **(E)** of SMC in response to 50 µM PGE_2_ (red) vs spontaneous activity (CTRL, black). **(F, G)** Percent of active cells after stimulation with PGE_2_, expressed relative to all morphologically detected SMCs **(F)** and relative to cells showing spontaneous activity prior to stimulation **(H)** Localized contractions triggered by PGE_2_, expressed as relative number of preparations. Calcium imaging data **(A–G)** are pooled from 218 oscillations, 109 SMCs, 13 preparations, and 5 mice. Contraction analysis **(H)** was performed on 10 preparations that retained a contractile response to the final CCh application (5 mice). ****p<0.0001 (Mann-Whitney test).

Bladder SMCs displayed spontaneous activity in the form of short transient increases in [Ca^2+^]_i_ (**Figure 2A**). The degree of the spontaneous activity was variable, with some SMCs showing little or no activity and others exhibiting more frequent events. The raw traces were binarized by detecting individual [Ca^2+^]_i_ oscillations and presented as raster plots in **Figure 2B**. The binarization was used to calculate parameters describing SMC activity (**Figures 2C, D**): active time, duration and frequency of [Ca^2+^]_i_ oscillations as well as percentage of active cells. Active time is widely used index quantifying cell activity on the range of 0 to 100% (Serdinšek et al., 2020; Marolt et al., 2022). In a pilot study we determined 50 µM PGE_2_ concentration as effective (**Supplementary Figure 2**) and was used in subsequent experiments. Application of 50 µM PGE_2_ effectively increased SMCs activity. This increase was evident as a higher overall active time, which increased from median 2.1% in control conditions to 6.3% during PGE_2_ stimulation (1^st^ quartile/median/3^rd^ quartile Q1/Me/Q3: 1.0/2.1/5.0% vs. 2.6/6.3/13.5%, p < 0.0001, N = 109 SMCs for CTRL and 109 SMCs for PGE, Mann–Whitney test; **Figure 2C**). The increased activity was mainly due to increase in oscillatory frequency rather than changed duration. More specifically, the frequency increased from 1.0 to 3.5 per minute (Q1/Me/Q3: 0.7/1.0/2.3 min-1 vs. 1.5/3.5/7.0 min-1, p < 0.0001; N = 96 SMCs for CTRL and 104 SMCs for PGE, Mann–Whitney test; **Figure 2E**). In contrast, duration of oscillations was not affected by PGE_2_ (Q1/Me/Q3: 1.050/1.052/1.052 s for controls vs. 1.050/1.052/1.052 s for PGE_2_ stimulation, p = 0.068; N = 96 SMCs for CTRL and 104 SMCs for PGE, Mann–Whitney test; **Figure 2D**).

Important aspect is to measure how many SMCs were recruited to the more active state by PGE_2_. To this aim, SMCs were classified as activated by PGE_2_ when their active time during stimulation exceeded the preceding control period. Based on this criterion, the PGE_2_ recruited 28% of SMCs that were morphologically identified as SMC (Q1/Me/Q3: 18/28/37%; N = 13 preparations; **Figure 2F**). Because morphologically identified cells that remained inactive throughout the protocol could not be verified as functionally responsive SMCs, we also expressed the number of PGE_2_-activated cells relative to active SMCs in the prestimulatory period based of change in active time (see methods for details). Using this approach, PGE_2_ activated all SMCs (Q1/Me/Q3: 100/100/100%; N = 13 preparations, **Figure 2G**). In addition to increasing [Ca^2+^]_i_ activity at the single-cell level, PGE_2_ also triggered localized tissue contractions, indicating that the calcium response was functionally coupled to mechanical output. These contractions remained spatially restricted. Whereas calcium imaging data are based on 13 preparations, contraction data are based on 10 preparations. Preparations that did not retain a contractile response to the final carbachol (CCh) application were excluded from the contraction analysis. Localized contractions were detected in 6 of 10 preparations (60%, **Figure 2H**).

### Effect of PGE_2_ on CCh responsiveness

**Figure 3:**
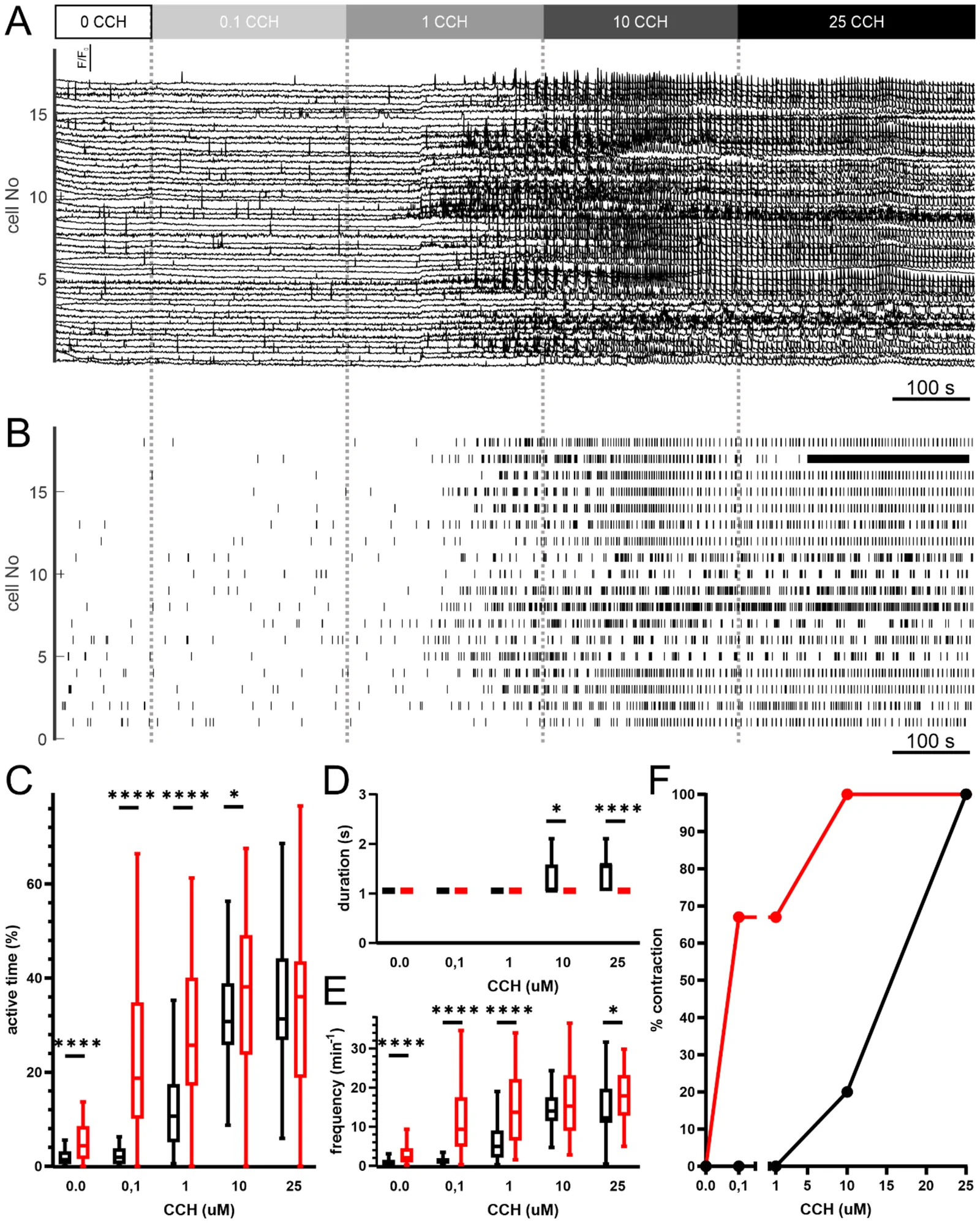
PGE_2_ resulted in a left shift of dose response curves to CCh. **(A, B)** Representative response of SMCs from the same bladder to increasing concentrations of CCh alone (0, 0.1, 1, 10, and 25 µM), shown as raw Ca²^+^ recordings **(A)** and corresponding binarized activity **(B)**. The stimulation protocol is depicted above the traces. **(C–E)** Active time **(C)**, duration **(D)**, and frequency **(E)** of SMCs during the CCh staircase-like protocol (CTRL, black) and with addition of 50 µM PGE_2_ (red). **(F)** Detected tissue contractions as a function of CCh concentration, expressed as percentage of tissue preparations. Pooled data from 700 oscillations, 140 SMCs, 9 preparations and 4 mice. *p<0.05, ****p<0.0001 (Mann-Whitney test).

To assess whether PGE_2_ modified responsiveness of bladder SMCs, we applied increasing concentrations of CCh either alone or in the presence of 50 µM PGE_2_ (**Figure 3**). Increasing the concentrations of CCh in the range from 0.1 to 25 µM resulted in progressively increasing SMC activity (**Figures 3A-E**), and a qualitatively similar concentration-dependent increase was observed when 50 µM PGE_2_ was added. However, in the presence of PGE_2_, comparable levels of activity were reached already at lower CCh concentrations, indicating that comparable levels of activity were reached at lower CCh concentrations in the presence of PGE_2_. More specifically, active time increased with increasing CCh concentration and reached a plateau of about 30% at 10 µM CCh, and PGE_2_-induced left shift was evident as increased active time up to plateau CCh concentrations (Q1/Me/Q3: 1.6/4.4/8.5% vs. 0.5/1.4/3.1% at 0 µM, p < 0.0001; 10.2/18.7/34.8% vs. 0.5/1.9/3.8% at 0.1 µM, p < 0.0001; 17.2/25.7/40.0% vs. 5.2/10.7/17.4% at 1 µM, p < 0.0001; and 23.8/38.1/49.1% vs. 25.8/30.7/38.8% at 10 µM, p = 0.0366, N = 88 SMCs for CTRL and 44 SMCs for PGE, Mann-Whitney test; **Figure 3C**). This effect was exerted mainly by frequency modulation rather than modulation of oscillatory duration. In control conditions, frequency increased from about 1 min^-1^ at 0 µM CCh to about 14 min^-1^ at 10 µM CCh, and addition of PGE_2_ resulted in larger frequency (Q1/Me/Q3: 1/2/4 vs. 0/1/1 min^-1^ at 0 µM, p < 0.0001; 5/9/17 vs. 1/1/2 min^-1^ at 0.1 µM, p < 0.0001; 7/14/22 vs. 2/5/9 min^-1^ at 1 µM, p < 0.0001; 9/15/23 vs. 12/14/17 min^-1^ at 10 µM, p = 0.0961; and 13/18/23 vs. 11/12/20 min^-1^ at 25 µM, p = 0.0104; N = 79–88 SMCs for CTRL and 37–43 SMCs for PGE, Mann-Whitney test; **Figure 3E**). In contrast, duration remained largely unchanged across CCh concentration regardless of PGE_2_ presence (**Figure 3D**) (Q1/Me/Q3: 1.052/1.052/1.052 s vs. 1.052/1.052/1.052 s at 0 µM, p = 0.74; 1.052/1.052/1.052 s vs. 1.052/1.052/1.052 s at 0.1 µM, p = 0.508; 1.052/1.052/1.052 s vs. 1.052/1.052/1.052 s at 1 µM, p = 0.057; 1.052/1.052/1.578 s vs. 1.052/1.052/1.052 s at 10 µM, p = 0.0167; and 1.052/1.578/1.578 s vs. 1.052/1.052/1.052 s at 25 µM, p < 0.0001; N = 79–88 SMCs for CTRL and 38–43 SMCs for PGE, Mann-Whitney test; **Figure 3E**).

To assess whether the increase in [Ca^2+^]_i_ activity translated into stronger mechanical output, and specifically whether PGE_2_ enhanced CCh-induced contractions, we also quantified tissue contractions. In control conditions, CCh-dependent contractility was observed only at concentrations above 1 µM and was detected as an increasing proportion of preparations displaying CCh-induced contractions (20% at 10 µM CCh, and 100% at 25 µM CCh, N = 5 preparations, **Figure 3F**). In the presence of PGE_2_, the contractile response was shifted to lower CCh concentrations, with generalized contractions already observed in 67% of preparations at 0.1 µM CCh and in 100% of preparations at > 10 µM CCh, N = 4 preparations (**Figure 3F**). Unlike the localized contractions observed with PGE_2_ alone, combined stimulation with PGE_2_ and CCh evoked generalized contractions across the preparation.

## Discussion

### Principal findings

The present study confirms that PGE_2_ is a potent modulator of mouse detrusor smooth muscle activity in acute bladder tissue slices. Application of PGE_2_ increased spontaneous Ca^2+^ activity in individual SMCs, primarily by increasing oscillation frequency rather than oscillation duration, and this cellular activation was accompanied by localized tissue contractions. In addition, PGE_2_ enhanced responsiveness to cholinergic stimulation, shifting CCh-induced Ca^2+^ activity and contractile responses toward lower agonist concentrations. These findings identify PGE_2_ as a local excitatory modulator that increases detrusor excitability and lowers the threshold for cholinergic activation.

Our findings are consistent with previous work showing that exogenous PGE_2_ promotes spontaneous rhythmic activity in the mouse urinary bladder. Kobayter and colleagues demonstrated that PGE_2_ increases detrusor tone, enhances phasic contractions, and elevates the frequency of spontaneous Ca^2+^ transients in bladder smooth muscle, independently of efferent nerve activation (Kobayter et al., 2012). Our results build on these observations by showing that PGE_2_ not only increases Ca^2+^ activity but also recruits a greater proportion of smooth muscle cells into an active state. Moreover, the effect of PGE_2_ was expressed mainly as an increase in oscillation frequency rather than duration. This is an important finding because increasing the frequency of Ca^2+^ oscillations can strengthen excitation-contraction coupling and promote contractile activity without the need for sustained elevation of intracellular Ca^2+^ in individual SMCs (Somlyo and Somlyo, 2003).

### Potential physiological mechanisms

The present study was not designed to identify the molecular mechanisms underlying the observed physiological responses. Nevertheless, previous studies suggest that PGE_2_ may increase detrusor excitability through activation of EP receptors, modulation of membrane excitability, and regulation of ion channels involved in Ca²^+^ entry (Schröder et al., 2004; Kobayter et al., 2012; Xue et al., 2013). Our findings are consistent with such mechanisms but do not distinguish between them. Future studies combining receptor-selective pharmacology, electrophysiology, and direct measurements of intracellular Ca²^+^ handling will be required to determine the pathways responsible for the interaction between PGE_2_ and cholinergic stimulation.

A strength of the present study is the application of quantitative single-cell Ca²^+^ analysis in an acute mouse detrusor tissue preparation to compare cholinergic responses in the absence and presence of PGE_2_. The preparation, previously established by Serdinšek et al. (2020), enables simultaneous analysis of Ca²^+^ event activity in many individual detrusor smooth muscle cells while preserving local tissue organization. This allowed us to relate changes in single-cell Ca²^+^ event activity to visually detected tissue movement and to determine how ongoing PGE_2_ exposure modifies responses to increasing concentrations of CCh. Using this preparation, we observed that PGE_2_ alone induced localized contractions, whereas combined stimulation with PGE_2_ and CCh produced generalized contractions across the preparation. This distinction suggests that PGE_2_ by itself is sufficient to increase local excitability and trigger focal mechanical responses, but that cholinergic drive is required for broader synchronization and stronger contractile recruitment of the tissue.

The leftward shift of the CCh response in the presence of PGE_2_ is one of the most relevant findings of this study. In practical terms, PGE_2_ enabled lower concentrations of CCh to evoke levels of Ca^2+^ activity and contractility that otherwise required stronger muscarinic stimulation. This pattern is consistent with enhanced responsiveness to cholinergic stimulation but does not establish receptor-level sensitization. Such sensitization could arise at several levels, including enhanced membrane excitability, facilitation of intracellular Ca^2+^ release, or modulation of receptor-coupled signaling pathways. Although the present experiments were not designed to resolve the underlying receptor mechanisms, the data are consistent with PGE_2_ increasing baseline detrusor excitability, thereby reducing the additional cholinergic input required to evoke detectable Ca²^+^ activity. Although PGE_2_ is generally considered to act through EP receptors, accumulating evidence indicates that prostaglandin-mediated bladder contractility may also involve FP receptors. In porcine bladder tissue, Stromberga et al. demonstrated that pharmacological blockade of FP receptors significantly attenuated both PGE_2_- and PGF_2_α-induced contractile responses, whereas selective EP receptor antagonists had little effect, suggesting that FP receptors can substantially contribute to prostaglandin-induced contractility in this preparation (Stromberga et al., 2020). Because receptor-selective pharmacology was not performed in the present study, we cannot determine which prostanoid receptor subtypes mediated the observed effects of PGE_2_ in mouse detrusor smooth muscle.

Although the present experiments were not designed to identify the EP (E-prostanoid) receptor subtype responsible for the observed effects, previous studies suggest several plausible mechanisms. PGE_2_ acts through four EP receptor subtypes with partly opposing actions. EP1, which is generally coupled to Gq signaling, is a strong candidate for excitatory effects in detrusor SMCs because it converges with the muscarinic M3 pathway involved in voiding contractions (Schröder et al., 2004). EP3 may also facilitate excitability through Gi-mediated inhibition of cAMP production, whereas EP2 and EP4 are more commonly linked to Gs/cAMP-dependent relaxant pathways, although EP4 may also contribute to inflammatory and afferent bladder signaling. Thus, the net effect of PGE_2_ on detrusor excitability likely reflects the balance between EP1/EP3-mediated excitation and EP2/EP4-mediated relaxation or sensory modulation (Hata and Breyer, 2004; Xue et al., 2013; Hou et al., 2021). The present findings provide a physiological framework for such future mechanistic studies by establishing how exogenous PGE_2_ modulates Ca²^+^ event activity and cholinergic responsiveness in the intact detrusor preparation.

Although PGE_2_ is primarily associated with EP receptors, FP receptors may also contribute to bladder responses. In porcine bladder preparations, PGE_2_ and PGF_2_α modulated urothelial, lamina propria, and detrusor contractility through FP receptors (Schröder et al., 2004). Because receptor-selective pharmacology was not performed in the present study, a contribution of FP receptors cannot be excluded.

At the ion-channel level, the increased oscillation frequency observed here may reflect enhanced membrane excitability and Ca²^+^ entry. PGE_2_-induced phasic activity in mouse bladder depends on L-type Ca²^+^ channels (Ferguson et al., 1997) and in guinea pig detrusor SMCs PGE_2_ inhibits large-conductance Ca²^+^-activated K^+^ channels, which would favor depolarization and increase L-type Ca²^+^ channel opening (Marolt et al., 2022). Because BK channels are key negative regulators of urinary bladder smooth muscle excitability, their inhibition provides a plausible explanation for the frequency-based increase in Ca²^+^ oscillations observed in the present study (Parajuli et al., 2014; Petkov, 2014). Together, these findings suggest that PGE_2_ may increase detrusor excitability by reducing K^+^-dependent membrane stabilization and promoting voltage-dependent Ca²^+^ entry (Kobayter et al., 2012; Parajuli et al., 2014).

### Physiological and clinical relevance

These findings are physiologically important in the context of bladder wall signaling. The urothelium and suburothelial compartment are now recognized as active sensory and signaling structures that release mediators, including prostaglandins, ATP, nitric oxide, and acetylcholine, in response to stretch and chemical stimulation (Birder et al., 2012). During bladder filling or inflammation, locally produced PGE_2_ may therefore act as a paracrine signal that increases detrusor excitability and facilitates communication between mucosal signaling pathways and the contractile apparatus. Although the urothelium was removed in our preparation, response to exogenous PGE_2_ indicates that detrusor tissue itself is competent to respond directly to this mediator.

From the clinical point of view, the possible role of PGE_2_ in bladder contractile dysfunctions such as overactive bladder (OAB) and underactive bladder (UAB) have been investigated in the last decades. It is well established that the release of prostaglandins has a direct influence on the micturition reflex. Prostaglandin overproduction has been observed in conditions such as bladder outlet obstruction, bladder overactivity due to spinal cord injury, and inflammation (Stromberga et al., 2020). It has been proposed that PGE_2_ contributes to pathogenesis of both OAB and UAB as well as bladder pain syndrome (BPS). BPS, also known as interstitial cystitis (IC), is a complex chronic medical condition that appears to affect the urinary bladder with symptoms of discomfort, pressure, or pain (Lim et al., 2026). Increased urinary PGE_2_ levels have been reported in patients with overactive bladder or BPS, suggesting enhanced prostaglandin signaling in the diseased bladder wall (Kim et al., 2006; Chen et al., 2024). A clinical study from Kim et. al., 2006 found significantly elevated urinary nerve growth factor (NGF), PGE_2_, and PGF_2α_ in women with OAB compared to control group (Kim et al., 2006). Of all investigated prostaglandins, PGE_2_ is thought to be most plausible contributor to bladder disorders, as it modulates bladder function via afferent signaling, induces overactivity of detrusor muscle by sensitizing capsaicin-sensitive afferent nerve endings, and is produced locally as a response to inflammation and distension (Stromberga et al., 2020).

The observed PGE_2_-induced sensitization in our study may therefore be relevant for pathological states characterized by urgency and detrusor overactivity. Within that context, our data provide a cellular mechanism that could contribute to symptom generation: elevated PGE_2_ may increase spontaneous detrusor activity and simultaneously reduce the amount of cholinergic drive required to trigger coordinated contractions. Such a combination would be expected to favor inappropriate bladder activation at lower filling volumes and may contribute to urgency-associated motor instability. This is in concordance with findings of some other studies in rabbit and guinea-pig animal models, which observed that detrusor SMCs contractions in response to acetylcholine and ATP are enhanced by prostaglandins (Anderson, 1982; Nile et al., 2010). This further implicates complex interactions between those mediators, which could potentially cause amplification of molecule signaling and increased detrusor contractility (Stromberga et al., 2020).

On the contrary, the role of prostaglandins in pathogenesis of UAB is not that well investigated despite the fact that some clinical studies have shown promising results in UAB treatment with combination of intravesical prostaglandin E2 and oral cholinergic bethanechol chloride (Hindley et al., 2004; Dan et al., 2020). Several different factors contribute to UAB: brain and/or spinal cord dysfunction, afferent and efferent nerve dysfunction, and myogenic failure. These factors can be caused by disease and conditions such as bladder outlet obstruction, diabetes mellitus, Parkinsons disease, spinal stenosis, pelvic fractures, infectious neurological disorders, as well as aging (Aizawa and Igawa, 2017). Of all these conditions, aging has been shown to influence detrusor muscle contractions to prostaglandins, histamine, and serotonin, thus contributing to UAB occurrence (Phelps et al., 2023).

### Limitations and future directions

Several limitations of the present study should be considered. First, the experiments were performed in *ex vivo* mouse tissue slices, which preserve important local architecture but cannot fully replicate the intact neural, urothelial, and biomechanical environment of the urinary bladder (Hennig et al., 2023). Second, the urothelium was removed during preparation; this was advantageous for isolating direct detrusor-level responses to exogenous PGE_2_, but it also means that endogenous urothelial PGE_2_ release, urothelial-afferent communication, and mucosa-derived modulation of detrusor activity were not assessed directly (Munoz et al., 2010; Durnin et al., 2019; Heppner et al., 2024). Third, contractions were scored visually as localized or generalized rather than quantified by direct force measurements. Fourth, the study does not identify the EP (E-prostanoid) receptor subtype or intracellular signaling cascade responsible for the observed effects. Fifth, only a single concentration of PGE_2_ (50 µM) was investigated; therefore, concentration-dependent effects could not be assessed. Sixth, because Calbryte 520 AM fluorescence was not calibrated, the present recordings do not provide absolute resting or peak intracellular Ca²^+^ concentrations. Consequently, the study cannot determine whether PGE_2_ altered the amplitude of individual Ca²^+^ events. The conclusions are restricted to changes in event frequency, duration, active time, cellular recruitment, and associated tissue contraction. Finally, the study was performed in adult female mice only, so the extent to which the results generalize across sex, strain, or disease models remains to be determined. Taken together, these findings provide a physiological rationale for future studies investigating modulation of prostaglandin signaling and its interaction with cholinergic pathways in experimental models of bladder dysfunction. However, the present data do not support conclusions regarding the therapeutic efficacy of specific pharmacological interventions.

## Conclusion

In conclusion, PGE_2_ increases spontaneous Ca^2+^ activity in mouse detrusor smooth muscle cells, promotes localized tissue contractions, and enhances responsiveness to cholinergic stimulation. The effect of PGE_2_ is expressed predominantly through an increase in oscillation frequency and is functionally associated with a leftward shift of CCh-induced activation and contractility. These findings support a model in which PGE_2_ acts as a local excitatory modulator of the bladder wall, capable of increasing detrusor excitability and reducing the cholinergic input required to evoke coordinated Ca²^+^ activity and contraction. This mechanism may be particularly relevant in conditions associated with increased prostaglandin signaling, such as overactive bladder or bladder pain syndrome, and highlights PGE_2_-dependent pathways as potential targets for future studies of bladder dysfunction.

## Data Availability

The datasets presented in this study can be found in online repositories. The names of the repository/repositories and accession number(s) can be found in the article/**Supplementary Material**.
