# Supplementary material for: Prostaglandin E2 modulates contractility of mouse bladder smooth muscle cells
Source: Front Physiol. 2026 Aug 12;17:1910107. doi: 10.3389/fphys.2026.1910107 (PMC13506759; doi:10.3389/fphys.2026.1910107)
Supplement: Supplementary file 1 [file DataSheet1.docx]

***Supplementary Figure 1: Effect of increasing PGE₂ concentrations on SMC active time.*** *Active time of detrusor SMCs during a staircase-like stimulation protocol with 0, 0.1, 1, 10, and 50 µM PGE₂. Data are presented as box-and-whisker plots; N = 23 SMCs at each concentration, data pooled from 4 preparations from 3 mice. PGE₂ significantly affected active time (p = 0.0004, Kruskal–Wallis test). Dunn’s multiple-comparisons test identified significant differences between control and 1, 10, and 50 µM PGE₂. * p < 0.05, ** p < 0.01, *** p < 0.001 versus 0 µM PGE₂.*

**Results:**

In a pilot staircase experiment, PGE₂ significantly increased SMC active time across the tested concentrations (p = 0.0004, Kruskal–Wallis test; Supplementary Figure 1). Active time increased from Q1/Me/Q3 (1^st^ quartile/median/3^rd^ quartile): 0.29/1.17/2.92 % in the absence of PGE₂ to 0.88/2.62/3.51 % at 0.1 µM PGE₂, 2.62/3.49/6.12 % at 1 µM PGE₂, 1.76/4.39/7.43 % at 10 µM PGE₂, and 1.75/3.94/8.31 % at 50 µM PGE₂ (N = 23 SMCs at each concentration, data pooled from 4 preparations from 3 animals). Compared with baseline, active time was significantly increased at 1 µM PGE₂ (adjusted p = 0.0244), 10 µM PGE₂ (adjusted p = 0.0008), and 50 µM PGE₂ (adjusted p = 0.0039), but not at 0.1 µM PGE₂ (adjusted p = 0.9747; Dunn’s multiple-comparisons test). No significant differences were detected among the tested PGE₂ concentrations. These pilot data confirmed that 50 µM PGE₂ reliably increased SMC activity and supported its use in the subsequent experiments.
